# Cognitive control, emotional value, and the lateral prefrontal cortex

**DOI:** 10.3389/fpsyg.2015.00758

**Published:** 2015-06-02

**Authors:** Matthew L. Dixon

**Affiliations:** Department of Psychology, Cognitive Neuroscience of Thought Laboratory, University of British ColumbiaVancouver, BC, Canada

**Keywords:** cognitive control, value, prefrontal cortex, motivation, self-control, emotion

## Introduction

Cognitive control refers to the intentional selection of thoughts, emotions, and behaviors based on current task demands and social context, and the concomitant suppression of inappropriate habitual actions (Miller and Cohen, 2001). Common situations that require cognitive control include: studying for an exam while resisting the impulse to check Facebook; having fruit instead of dessert when on a diet; and being patient with one's kids instead of yelling at them for spilling juice on the carpet. Historically, there has been preferential interest in *how* cognitive control operates. An influential model suggests that the lateral prefrontal cortex (LPFC) represents rules or instructions in working memory and that this information adaptively guides perceptual and motor processing in posterior brain regions, thus resulting in the selection of appropriate behaviors, and the suppression of maladaptive habitual actions (Miller and Cohen, 2001; Bunge, 2004). Surprisingly, there has been less focus on *why* individuals choose to engage cognitive control in the first place (Dixon and Christoff, 2012; Botvinick and Braver, 2015). In this article, I outline a *value-based framework of cognitive control* which suggests that: (1) individuals choose to engage cognitive control when they expect that it will produce an emotionally valued outcome (i.e., a reward or the avoidance of punishment); (2) the LPFC is a critical neural substrate involved in representing the value of engaging cognitive control; and (3) the LPFC is organized along a rostro-caudal axis, with different sub-regions contributing to different elements of the decision to employ cognitive control.

## Cognitive control and emotional value

A number of studies have shown that individuals are naturally disinclined to engage cognitive control (McGuire and Botvinick, 2010; Dixon and Christoff, 2012; Botvinick and Braver, 2015). For example, when given the choice between two tasks, individuals will reliably choose the easier task (McGuire and Botvinick, 2010). It requires considerable effort to intentionally direct action, and this may often be experienced as aversive. This has led to the idea that cognitive control carries an intrinsic *effort cost* (Botvinick and Braver, 2015). Accordingly, individuals may only engage cognitive control if they think that it will produce an emotionally valuable outcome that outweighs this effort cost.

We examined this idea by offering participants the choice between engaging cognitive control or selecting a habitual action, and varying the amount of money they earned based on their choices (Dixon and Christoff, 2012). The results were striking: participants invariably selected the habitual action when it was expected to yield an equal or larger monetary reward. In contrast, participants frequently chose to engage cognitive control when it was expected to result in a larger monetary reward than the habitual action. This suggests that the anticipated emotional value of the monetary outcome was a critical factor influencing the decision of whether or not to engage cognitive control. This finding complements other work demonstrating that reward incentives often lead to faster and more accurate performance (Locke and Braver, 2008; Jimura et al., 2010; Padmala and Pessoa, 2011; Chiew and Braver, 2013; Etzel et al., 2015).

**Figure 1:**
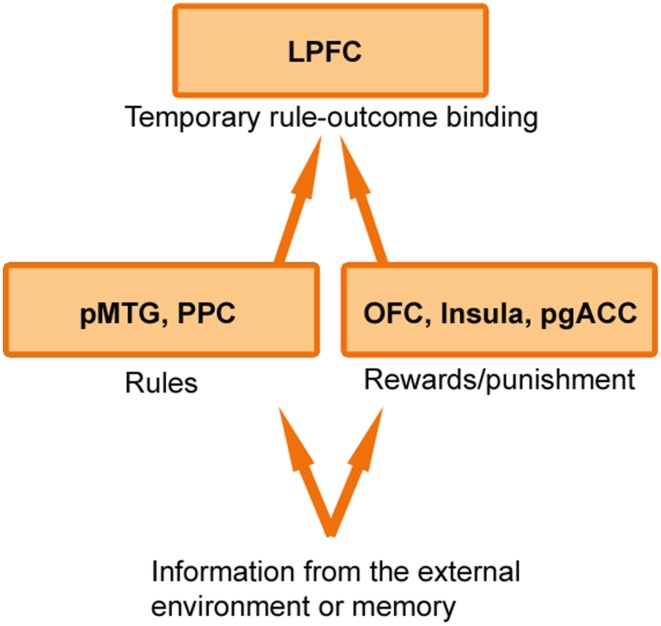
**Rule-outcome associations in the LPFC**. Information about rules may be first registered in regions including the posterior middle temporal gyrus (pMTG) and posterior parietal cortex (PPC), while information about rewards and punishment may be first registered in regions including the orbitofrontal cortex (OFC), insula, and pregenual anterior cingulate cortex (pgACC). This information may then be passed to the lateral prefrontal cortex (LPFC) where it is temporarily bound into a rule-outcome association that specifies whether engaging cognitive control is expected to result in an emotionally valued outcome. Other value regions including the ventral striatum and ventromedial prefrontal cortex may also interact with the LPFC in a context dependent manner.

While it is clear that cognitive control and emotional value systems do not operate independent of one another (Watanabe and Sakagami, 2007; Pessoa, 2008; Dixon and Christoff, 2014), the precise mechanism underlying their interaction remains to be determined. I suggest that the brain flexibly creates *temporary bindings or associations between rules for action that support cognitive control and the emotional outcomes that are expected to be obtained from rule-use* (Figure 1). When a rule becomes associated with a high-value outcome, this may sharpen and stabilize the rule representation in working memory (Etzel et al., 2015), thereby enabling individuals to hold this information in mind until it has successfully guided behavior. Thus, if a student is deliberating between going to the movies or studying for an exam, the student will be more likely to resist the immediate pleasure of going to the movies if they focus on the causal relationship between studying (which requires cognitive control) and the desired outcome it will produce (a good grade). If the student's attention is merely drawn to the effort of exerting cognitive control, they will be more likely to go to the movies. Similarly, when parents use the phrase “because I said so” after requesting their child to exert self-control, they should not be surprised when the child does not comply, because they have not provided the child with an incentive to invest the effort.

An important implication is that in some cases, individual and group differences in cognitive control may be erroneously attributed to differences in “cognitive” mechanisms (e.g., the ability to hold rules in mind, reasoning, response inhibition), when in fact it reflects differences in the perceived emotional value of engaging those cognitive mechanisms. Furthermore, there may be substantial individual differences in the *types of incentives* that effectively motivate cognitive control—some individuals may be more responsive to monetary rewards, others to food rewards, and others to social praise, intrinsic motivation, or the avoidance of punishment.

## The lateral prefrontal cortex and rule-outcome associations

Is there evidence that the brain flexibly creates temporary associations between rules that support cognitive control and the emotional value of expected outcomes? A number of studies have provided indirect evidence for this idea, showing that cognitive control related activation in the LPFC is amplified by reward incentives (Kouneiher et al., 2009; Beck et al., 2010; Jimura et al., 2010; Padmala and Pessoa, 2011). To directly examine this hypothesis, we had participants perform a cognitive control task in which they used one of two rules to respond to stimuli on each trial, and earned one of two monetary outcomes based on their performance (Dixon and Christoff, 2012). Using fMRI-adaptation, we looked for any regions that were sensitive to specific rule-outcome pairings. We found that the LPFC exhibited precisely this effect. Notably, the relevant rules and monetary outcome changed from trial to trial suggesting that the LPFC was continuously forming and then dissolving such associations in a flexible manner. In contrast to the LPFC, other value-related regions including the orbitofrontal cortex, anterior cingulate cortex, and ventral striatum were sensitive to the expected reward outcome, but did not encode rule-outcome associations. Recent theoretical work has suggested that the mid-cingulate cortex may play an important role in determining the value of engaging cognitive control (Shenhav et al., 2013), however, the findings from this study favor the interpretation that the LPFC is the critical region in determining when it is worth it to intentionally direct action. This idea is consistent with recent evidence demonstrating involvement of the LPFC in comparing the value of different actions (Morris et al., 2014). In particular, the LPFC has been implicated in “model-based” decision making—the construction of an internal model of the world, including the relationships between context, task-rules, and anticipated outcomes, in order to decide between choice options (Smittenaar et al., 2013; Buckholtz, 2015). Buckholtz (2015) has convincingly argued that the LPFC's capacity to construct rule-outcome associations in a model-based manner is a critical computation underlying adaptive social behavior.

How does the LPFC create rule-outcome associations? One possibility is that information about rules and information about reward/punishment is first registered in separate specialized posterior brain regions, and then is passed to the LPFC which creates a temporary association between this information within working memory (Figure 1). Consistent with this idea, we found that the LPFC exhibited *synchronized* activation with reward processing regions (e.g., orbitofrontal cortex, ventral striatum, insula, pregenual anterior cingulate cortex) and with rule processing regions (e.g., posterior parietal cortex, posterior middle temporal gyrus) when participants engaged cognitive control in order to obtain a monetary outcome (Dixon and Christoff, 2012). Extending these findings, recent work has shown that a frontoparietal network (including the LPFC) flexibly shifts connectivity patterns with other parts of the brain from trial to trial based on task demands (Cole et al., 2013). In line with these findings, LPFC neurons do not exhibit an intrinsic tuning toward a specific type of information, but rather, flexibly code information that is currently relevant on a given trial (Stokes et al., 2013). Thus, the adaptive coding properties and widespread anatomical connections of the LPFC may allow for the temporary construction of associations between diverse inputs, including rule and value information.

## Emotion regulation as value-based cognitive control

This value-based framework of cognitive control may provide a new understanding of how the LPFC contributes to emotion regulation. Emotion regulation often involves the effortful use of rules or cognitive strategies in order to alter one's emotional state (Ochsner and Gross, 2005; Gross, 2015). Traditionally, the LPFC has been linked to the implementation of these strategies, such as attentional distraction, reasoning about emotions, and reinterpreting the meaning of events (Ochsner and Gross, 2005). However, the LPFC may also play an important role in the decision to *initiate* emotion regulation. Here are two examples of how it may contribute: (1) it may be involved in determining when emotion regulation is necessary by representing the value of a current emotional state in relation to social rules (e.g., determining that it is not appropriate to feel/express anger because one is currently at a work meeting); and (2) it may be involved in determining the value of employing effortful regulation strategies by representing the relationship between a given strategy (e.g., reappraisal of an event's meaning) and a desired outcome (e.g., less anger). Thus, the decision to instantiate emotion regulation may depend on the flexible synthesis of rule-outcome associations. This perspective is compatible with Gross (2015) extended process model of emotional regulation, which emphasizes the role of second-order valuation mechanisms in triggering the engagement of emotion regulation strategies. The two examples noted above broadly correspond to the identification and selection stages of Gross' model, respectively.

## The hierarchical organization of the lateral prefrontal cortex

Accumulating evidence suggests that the LPFC is hierarchically organized along a rostral to caudal (anterior to posterior) axis (Christoff and Gabrieli, 2000; Koechlin et al., 2003; Petrides, 2005; Bunge and Zelazo, 2006; Badre and D'Esposito, 2009; Christoff et al., 2009; Dixon et al., 2014). I consider how this hierarchical organization relates to the decision of whether or not to engage cognitive control. The rostral LPFC (area 10), mid-LPFC (areas 9/46/45), and caudal LPFC (areas 44/8/6) are discussed in turn.

Cognitive control often operates in service of desired long-term outcomes (e.g., studying over the course of a semester in order to get an “A”). The rostral LPFC plays a role in meta-cognitive awareness (Fleming et al., 2010; McCaig et al., 2011; De Martino et al., 2013) and relational processing (Christoff et al., 2001; Wendelken et al., 2008). It may contribute to the decision to engage cognitive control by enabling individuals to reflect on their thoughts and feelings, and to establish long-term priorities by comparing the value of potential future outcomes. Consistent with this, the rostral LPFC is recruited when individuals plan steps to attain a future goal (Gerlach et al., 2014), choose to avoid situations that may interfere with the attainment of future rewards (Crockett et al., 2013), monitor progress toward a desired future reward (Dixon et al., 2014), and select actions directed toward future rather than immediate rewards (McClure et al., 2004; Jimura et al., 2013). This region may work in concert with the ventromedial prefrontal cortex, which is also involved in representing the affective value of future scenarios (D'Argembeau et al., 2008; Benoit et al., 2014; Gerlach et al., 2014). One possibility is that the VMPFC represents the value of potential future outcome, and the RLPFC contributes to the selection of one outcome to pursue as a goal. Interestingly, meditation practice is associated with increased gray matter volume in the rostral LPFC (Fox et al., 2014), suggesting that this meta-cognitive capacity to step back and reflect on thoughts (including desired future outcomes) can be trained and improved.

Whereas the RLPFC plays a role in decisions regarding overarching priorities, the mid-LPFC operates on a shorter time-scale, contributing to the decision of whether or not to employ rules to intentionally direct action at any given moment. This decision depends on discerning whether the outcomes expected from rule-use are sufficiently valuable to offset the effort cost. Thus, a critical component is constructing rule-outcome associations. Notably, our fMRI-adaptation study revealed robust encoding of rule-outcome associations in the mid-LPFC (Dixon and Christoff, 2012). Consistent with this, recent findings indicate that mid-LPFC activation reflects an interaction between the complexity of rules that are required to respond to stimuli and the size of an expected reward outcome, and this neural response correlates with individual differences in behavior (Bahlmann et al., 2015). Furthermore, mid-LPFC is reliably activated in studies of emotion regulation (Buhle et al., 2014), and may be involved in representing associations between rules/strategies (e.g., reappraisal) and desired outcomes (e.g., less sadness). The function of the mid-LPFC may depend on transient interactions with rule-processing and value-processing regions, and such network interactions may depend on context. The ventral striatum is activated during the anticipation of imminent rewards and the opportunity to exercise choice (Knutson et al., 2001; Leotti and Delgado, 2014), and may provide motivational signals to the mid-LPFC that promotes the engagement of cognitive control to obtain immediately available rewards. In contrast, the opportunity to make progress toward a desired future outcome may engage cognitive control via positive interactions between the rostral LPFC, mid-LPFC, and ventromedial prefrontal cortex, and inhibitory interactions between these regions and the ventral striatum (Diekhof and Gruber, 2010; Jimura et al., 2013; Van Den Bos et al., 2014).

Once the decision to engage cognitive control has been made, it requires that rules be translated into specific voluntary actions. The caudal part of the LPFC is involved in this process (Koechlin et al., 2003; Bunge, 2004; Petrides, 2005). The caudal LPFC is strongly activated during the execution of actions (Dixon et al., 2014), and facilitates the execution of context appropriate actions over competing actions by representing sensorimotor associations (Koechlin et al., 2003; Bunge, 2004; Petrides, 2005). Thus, caudal LPFC activity reflects the embodied output of the decision process—the behavioral instantiation of cognitive control.

## Conclusion

Considerable evidence suggests individuals will engage cognitive control when they expect that it will produce a valued outcome that outweighs the intrinsic effort cost—this may be an immediate or future reward, or a desired change in one's emotional state in the absence of external incentives. The LPFC contributes to this process, with the rostral LPFC representing desired long-term outcomes, the mid-LPFC supporting the flexible construction of rule-outcome associations, and the caudal LPFC translating rules into specific voluntary actions. The LPFC operates in the context of interactions with widely distributed rule and value-related regions (Dixon and Christoff, 2012, 2014). Much remains to be learned about the mechanisms underlying the decision to engage cognitive control, making this an important topic for future research.

### Conflict of interest statement

The author declares that the research was conducted in the absence of any commercial or financial relationships that could be construed as a potential conflict of interest.
